# Development of virtual ophthalmic surgical skills training

**DOI:** 10.1038/s41433-021-01896-1

**Published:** 2022-01-20

**Authors:** Chandni Gupta, Christin Henein, Christopher Ashton, Achini Makuloluwa, Rashmi G. Mathew

**Affiliations:** 1 University College London Institute of Ophthalmology, 11-43 Bath St, London, EC1V 9EL UK; 2 National Institute for Health Research Biomedical Research Centre for Ophthalmology, Moorfields Eye Hospital and UCL Institute of Ophthalmology, London, UK; 3 Moorfields Eye Hospital NHS Foundation Trust, London, UK; 4 Department of Undergraduate Medical Education, Moorfields Eye Hospital, London, UK

**Keywords:** Education, Outcomes research

## Abstract

**Background:**

This study aims to assess whether ophthalmic surgical skills can be taught successfully online to a diverse international and interprofessional student group.

**Methods:**

Mixed methods study involving 20 students and 5 instructors.

Each student completed a pre-session and post-session questionnaire to assess their perceptions regarding online instruction. Changes in questionnaire responses were analysed using Wilcoxon signed rank (SPSS 25). Semi-structured interviews were conducted to assess instructor perceptions towards virtual surgical skills teaching. Thematic analysis was undertaken using NVivo 12.0 software.

**Results:**

There was a 100% completion rate of pre- and post-session questionnaires.

Prior to the session, lack of instructor supervision and inability to provide constructive feedback were emergent themes from students. Pre-session concerns regarding online delivery: 40% of students thought their view of skills demonstration would be negatively impacted, 60% their level of supervision and 55% their interaction with instructors. Following the session 10%, 15% and 5% held this view respectively. All students were ‘satisfied’ or ‘very satisfied’ regarding the ‘Surgeon’s View’ camera angle as well as the use of breakout rooms. 75% perceived an improvement in their confidence in instrument handling, 80% in cable knot tying and 70% in suture tying.

Overall student rating for the virtual surgical skills session was 8.85 (±1.19) out of 10 (10 being most satisfied).

**Conclusions:**

We demonstrate that successful delivery of a virtual ophthalmic surgical skills course is feasible. We were able to widen accessibility and participation through virtual delivery, which has future implications for ophthalmic surgical teaching and its reach.

## Introduction

The Covid-19 pandemic has not only hindered ophthalmic residency training and progression; it has also disrupted vocational university courses such as medicine and dentistry [1, 2]. Students have had to contend with reduced face-to-face teaching; this includes lectures, simulation sessions and clinical placements [2]. Lecture-based teaching has been successfully transferred online via virtual platforms such as Blackboard Collaborate®, Microsoft Teams® and Zoom® [3]. Simulation and clinical bedside teaching however, have not been as readily transferable to the online setting due to lack of accessibility of equipment, face-to-face instruction and direct patient contact [4, 5].

Simulation is associated with large gains in knowledge, skills, behaviours and is therefore a mainstay of both undergraduate and postgraduate medical education [6, 7]. Non-essential travel and social distancing guidance have meant that surgical skills simulation practicals have had to be cancelled. Recently some educators have shifted these face-to-face sessions online [8].

The University College London (UCL) Ophthalmology Masters programme offers basic ophthalmic surgical skills training sessions as part of the curriculum, to an inter-professional group of students. Prior to the pandemic, students from all over the world would attend this course in person. Historically, the glaucoma module surgical skills session would take place in a dedicated dry lab. Since August 2020, tutors were asked to adapt their teaching so that it could be delivered exclusively online.

### Aims

This study aims to assess whether ophthalmic surgical skills can be taught successfully to a diverse interprofessional student group via virtual platform.

## Materials & methods

### Preparation

In preparation for the virtual surgical skills session, students were sent ophthalmic surgical kits in advance. Each kit contained: three suture packs, toothed forceps, Castroviejo needle holder and Westcott scissors. Students were emailed instructions prior to the session which included which fruits to use for the practical, how to prepare them and what to expect on the day.

Overall five instructors were involved in the delivery of the practical session. All instructors were in their fourth year of ophthalmic residency training or above, and competent in ophthalmic surgical skills. Instructors were also emailed instructions regarding the session and links to pre-recorded surgical skills videos. A pre-session briefing was held with all instructors via Zoom®. As part of the briefing, instructors were shown how to navigate the platform; including use of breakout rooms, as well as how to demonstrate the taught skills in ‘Surgeon’s view’.

**Fig. 1:**
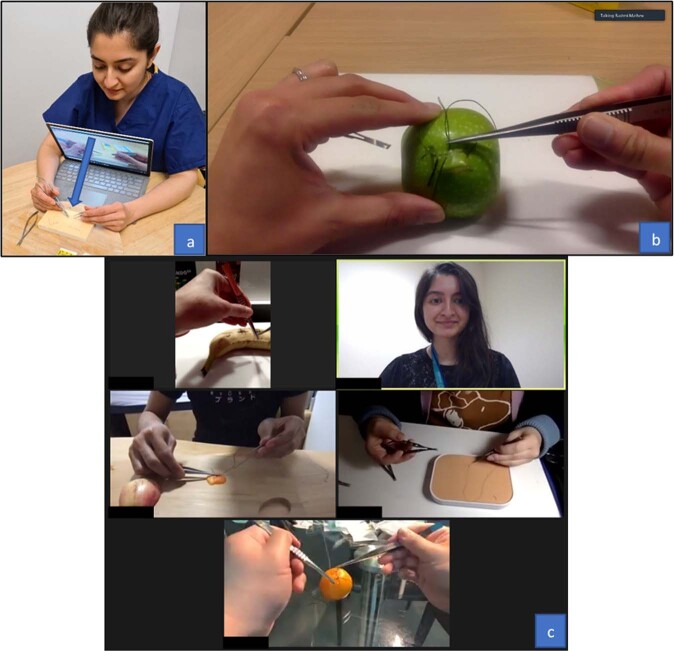
Use of ‘Surgeon’s View’ and Breakout Rooms. **a** How to achieve ‘Surgeon’s view’ by turning the camera device and angling the camera angle (arrow). **b** Surgeon’s view of trabeculectomy releasable suture demonstration. **c** Instructor’s view whilst supervising students in the breakout room.

‘Surgeon’s view’ is a specific camera angle enabling viewers (in this case students) to see exactly what the surgeon sees. It reduces cognitive overload as the student is able to directly emulate the steps of the skill rather than needing to mentally invert the process for delivery. ‘Surgeon’s view’ was achieved by turning our camera devices 180 degrees and angling the camera angle so that the fruit and our hands were in full view (Fig. 1a and b).

Videos were created in ‘Surgeon’s view’ specifically for the session and could be accessed via YouTube® links. Instructional videos were first made showing how to perform reef and slip knots using a cable so that students could understand how to tie a knot on a macro level. Further instructional videos were created demonstrating suturing and knot tying using sutures and surgical instruments on the prepared fruit. A final video was made showing how to tie a releasable suture as part of trabeculectomy surgery [9].

### Surgical skills teaching delivery

The Zoom® platform was chosen over other platforms to deliver the virtual surgical skills session due to its reliability and accessibility in countries outside of the United Kingdom. Synchronous teaching began with a PowerPoint presentation describing suture materials, instrument and suture handling in the main virtual teaching room. For the practical skills element, students began by watching a pre-recorded video on how to tie a reef knot with a cable, after which they were assigned to breakout rooms where they could practice this skill under direct supervision of an instructor (Fig. 1c). This was practiced until students had obtained a level of proficiency. This cycle was repeated for each of the remaining skills starting with basic general surgical skills and progressing to advanced ophthalmic skills: tying a slip knot with a cable, suturing and tying a reef knot, suturing and tying a slip knot, tying a trabeculectomy releasable suture. Instructor to student ratio in the breakout rooms was 1:4. At the end of the session, students were again brought back to the main teaching room for a PowerPoint presentation summarising key points of the day and reflecting on learning outcomes.

### Data collection

This is a mixed methods study where both qualitative and quantitative data were collected to assess student perceptions. Semi-structured interviews were used to understand instructor perceptions towards the virtual surgical skills session delivered.

### Student perceptions

Students were sent pre-session and post-session questionnaires via Google® Forms. The pre-session questionnaire consisted of seven Likert questions and one open-ended question. Students were asked about their baseline surgical skills, their perceptions regarding a virtual ophthalmic surgical skills course and to rate their confidence in surgical instrument handling, basic suturing capabilities and knot tying. In addition, they were asked whether their view, level of supervision or interaction with tutors would be negatively impacted by holding the session virtually.

The post-session questionnaire consisted of 17 Likert type questions and two open-ended questions about equipment, virtual platform, pre-recorded skill demonstration videos, practical session and supervision (see supplementary material).

To measure internal consistency of pre-session and post-session questionnaires Cronbach alpha (α) was calculated using SPSS 25 software. Internal consistency measuring *α* ≥ 0.7 was considered acceptable (pre-session questionnaire *α* = 0.72, seven items and post-session questionnaire *α* = 0.72, 12 items).

### Instructor perceptions

Semi-structured interviews were conducted to evaluate instructor perceptions towards the virtual session and consisted of 12 core questions (see supplementary material). All five instructors were interviewed by an independent interviewer. Interviews were digitally recorded and transcribed verbatim, before undertaking thematic analysis.

### Data analysis

Changes in pre-session and post-session responses (six items) were analysed using Wilcoxon signed rank (SPSS 25). The remainder of items were summarised using descriptive and summary statistics. Thematic analysis was used to evaluate the answers to open-ended questions from students and instructors. An inductive analytic approach based on the constant comparative method was used for coding, aggregation, and theme development. The qualitative analysis was done using NVivo 12.0 software.

## Results

Of the 21 students enroled on the MSc programme 2020/2021, 20 students participated in the virtual surgical skills session. All students completed the pre- and post- session questionnaires. There was a preponderance of female (57%), postgraduate (67%) and students studying within the UK (67%) (see Table 1). 25% of students had previously attended a face-to-face surgical skills course.

**Table 1:** Demographics of students who participated in the virtual surgical skills session.

Student Demographics	
Age (years)	Median 24, range (22–40)
Gender	Female 57% (*n* = 12)
Nationality	UK 33%, (*n* = 7)
	Asia 33%, (*n* = 7)
	Europe 14%, (*n* = 3)
	Middle East 10%, (*n* = 2)
	South America 5%, (*n* = 1)
	Africa 5%, (*n* = 1)
Location of study	UK 67% (*n* = 14)
	Outside UK 33% (*n* = 7)
Professional background	Medical student 33% (*n* = 7)
	Qualified Optometrist 24% (*n* = 5)
	Qualified Nurse 5% (*n* = 1)
	Qualified doctor 38% (*n* = 8)
Years post qualification where applicable	Median 3.5, range 1–15 years
	(excludes medical students)
Previous attendance to surgical skills course	Yes 25% (*n* = 5) • Virtual: 0 • Face to Face: 25% (*n* = 5) • Both: 0

### Pre-session student concerns

Thematic analysis of open-ended questions regarding pre-session student concerns were examined and themes of instructor supervision, feedback and surgical kit emerged. In particular, there were concerns regarding the ability of instructors to guide students through a skill, student ability to clearly view live demonstrations, as well as the instructor’s view of students performing the task.

*“It could be difficult to supervise my technique, i.e. how I’m using my hands, the amount of pressure used, which way around I’m tying the sutures”*

*“Not getting a good view. Not getting feedback. Not being able to properly show what I’m doing”*.

### Pre-session instructor concerns

Thematic analysis highlighted difficulty in articulation of instructions, visualisation and instructor to student ratio as possible challenges perceived by instructors in holding the skills session online.

*“I had a couple of concerns … the quality of the video … [and] not being able to visualise what the student is doing.”*

*“The main concern that I had was being able to describe everything adequately and not being able to show students or have that tactile element of teaching.”*

### Student confidence in surgical skills and attitude towards virtual surgical skills session

**Fig. 2:**
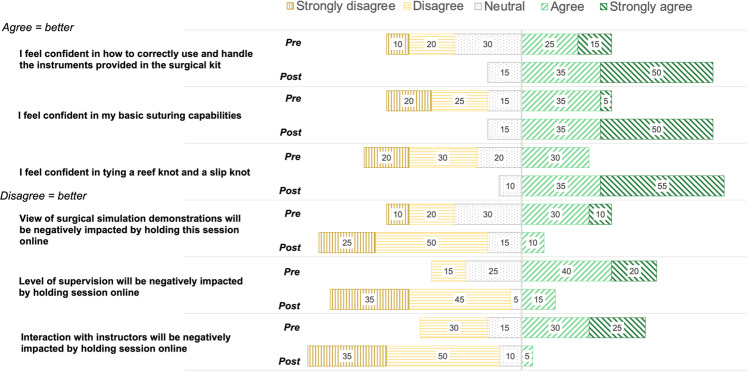
Student Responses. Pre- and post-session student responses regarding perceived confidence in surgical skills and attitude towards the virtual surgical skills session. *n* = 20, Wilcoxon signed ranks test *p* < 0.05 for all six questions.

Pre- and post-session questionnaires were used to assess changes in student reported confidence in surgical skills and attitude towards virtual surgical skills training (Fig. 2). At baseline 40% of students ‘agreed’ or ‘strongly agreed’ with the statements “I feel confident in how to correctly use and handle instruments” and “I feel confident in my basic suturing capabilities” and 30% ‘agreed’ or ‘strongly agreed’ with the statement “I feel confident in tying a reef knot and slip knot”. Overall, 75% perceived an improvement in their confidence in instrument handling, 80% in cable knot tying and 70% in suture tying. Following the session, the majority of students disagreed that virtual delivery of surgical skills training negatively impacted their view of live demonstrations, interaction with instructors or the level of supervision they received.

**Fig. 3:**
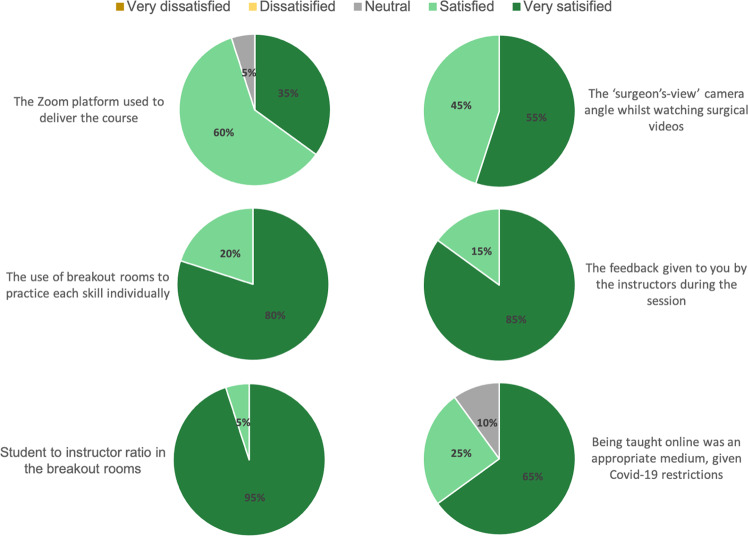
Student Responses. Student responses regarding satisfaction with virtual delivery of surgical skills session.

Overall rating for the virtual surgical skills session was 8.85 (±1.19) out of a scale from 0 to 10 (10 being most satisfied). Questions regarding delivery of teaching via the virtual platform are summarised in Fig. 3. All students were ‘satisfied’ or ‘very satisfied’ with the breadth and depth of the content covered and the subject knowledge and enthusiasm of the instructors, as well as their ability to ask questions via the platform. All students were ‘satisfied’ or ‘very satisfied’ with the balance of differing teaching methods used (lecture, video, breakout room), the lecture content, the video demonstrations and all the practical skills sessions. One student was ‘dissatisfied’ with the surgical equipment provided.

### Post-session student feedback themes

Thematic analysis of open-ended questions regarding student feedback on what went well was examined and themes of practicals in breakout rooms, supervision and individualised feedback emerged.

*“Most enjoyable session of the course so far. I liked that we had instructors with us in the breakout rooms to ask about any steps we were unsure about. Also, the demonstration videos beforehand were very useful.”*

*“The approach going from bigger to smaller” [starting from cable tying to demonstrate knot configuration before moving to suture tying, and finally trabeculectomy releasable suture tying]*

### Post-session instructor feedback themes

Emergent themes were teaching flexibility, visualisation, clarity of instruction, accessibility and personalised learning.

#### Teaching flexibility

Teaching flexibility emerged as a theme with regards to session preparation and decision-making. Additional resources to supplement the session were created such as the instructional videos and pre-course reading material. Flexibility was also mentioned in the context of adapting the pace of learning for students and modifying the skills covered in the allotted time. For instance, the schedule and content were revised to accommodate different learning styles and paces, and to also take into account the challenges that online facilitation may present.

*“A lot of additional planning that we had to do in the lead up to [the session]: create instruction sheets for students; create instruction sheets for tutors; we had a briefing session for all tutors to make sure that everybody knew and understood how to teach; I created some videos for the students which were done in surgeon’s view”*.

#### Visualisation

Instructors reported on the inability to view students’ non-verbal cues whilst they were practising the surgical skills, and how this reduced the human factor of teaching. Furthermore, whilst the instructor was demonstrating the skill in ‘Surgeon’s View’ they were unable to view their student group. A suggested solution was to use an adjustable web camera so that the instructor can demonstrate and view the panel of students simultaneously.

*“You need [webcam] setup. You could point the webcam away from you but still see the screen. That is what I would change for the next session.”*

#### Clarity of instruction

Instructors commented on the need for clear and concise teaching instructions when leading the break-out group practicals; they could not rely on tactile feedback and non-verbal communication, which would have occurred during a face-to-face session.

*“I felt that I had to make sure that my instructions were a lot clearer because there wasn’t the ability to use tactile cues to assist the students.”*

Two instructors suggested capping instructor-to-student ratio (to four students) in each breakout room and to reconvene with the same instructor for each practical to build relationships with students and gain a better understanding of their learning needs in order to better support them. Instructors also reported a relationship between student motivation and student acquisition of skills.

*“If you have the same group you build up a bit more rapport and [students] get to know you.”*

*“[Acquisition of skills] depended on how motivated each candidate was. Some were very motivated and got on with the activity and acquired the skills but some were maybe not motivated enough or maybe feeling frustrated that they were not getting it, and it was difficult for me to motivate them virtually.”*

#### Accessibility and individualised learning

Students were able to attend and engage in the session remotely, even those who were not based in the UK. An important feature of online learning is that the view of the demonstration is the same for all students, which is not always possible in face-to-face teaching.

*“Because we created videos for them to watch, and as they can ‘pin’ my video when I’m doing live demonstrations, the view is much more equitable for everyone.”*

*“It meant that fewer students were left out…we were able to have a global reach with many of the [students] taking the module outside of the UK.”*

*“[Students] were provided with equipment and resources to keep at home and so they have the opportunity for more hours of practise.”*

Individualised learning describes an instructor’s ability to respond to an individual student’s learning needs. This was achieved with the use of the ‘pin video’ function on Zoom® to enlarge the view, and observe and instruct students one at a time. A student’s individual concerns and identified learning needs were more easily addressed in small group breakout practical sessions.

*“Having breakout room facility [was comparable to face-to-face instruction] to help students on a more individual basis, and to help them acquire the skills.”*

## Discussion

The UCL Ophthalmology Masters curriculum was transferred to predominantly online teaching in response to the COVID-19 outbreak. Lecture-based teaching was easily transferable for online delivery however, virtual teaching of clinical and surgical skills sessions required significant pedagogical consideration and planning. A recent editorial stressed the importance of in-person ophthalmic simulation training for maintenance of surgical skills and competence [10]. Here we discuss the findings from our virtual ophthalmic surgical skills session and the purposeful steps taken to ensure successful online delivery to a diverse interprofessional student group. We note that these steps can also be utilised to teach generic surgical skills and are not exclusive to ophthalmic surgical skills training. General tips for course preparation and delivery are outlined in Table 2.

**Table 2:** Virtual ophthalmic surgical skills session, 10 top tips.

1	Surgical equipment and resources (e.g. pre-recorded videos and instructions) provided to students and instructors well in advance of session
2	Multi-modal learning resources cater better for different learning styles
3	Provide instructions on set-up of camera, including virtual platform settings (default video mirroring)
4	Schedule session taking into account student timezones
5	Ensure good internet connectivity and speed
6	Whole group demonstrations should precede breakout room practical session
7	Limit instructor to student ratio in breakout rooms (in our case 1:4)
8	Use of ‘pin video’ facility to observe individual students performing their practical skill and providing individualised feedback
9	Use a dark multi-braided suture (e.g. black dyed silk) and a light coloured fruit to provide contrast and enhance student and instructor viewing of skills practice and demonstrations
10	Maintain a separate communication channel amongst instructors. Particularly useful in breakout groups to ensure skills practice sessions run smoothly and are completed at roughly the same time.

Overall, students rated the session 8.85 (±1.19) out of a scale from 0 to 10 (10 being most satisfied). Student perceptions of their ability to interact with tutors, level of supervision and view of demonstrations positively changed following the teaching session, as did their confidence in instrument handling, knot tying and suturing. In addition, students could continue to practice the skills after their session, as they had the surgical kit with them at home.

95% of our students were ‘very satisfied’ or ‘satisfied’ with the Zoom® platform used to deliver the teaching session. Other medical educators have also successfully used this platform to deliver surgical skills teaching [11, 12]. For our session the Zoom® platform was used to deliver presentations, play instructional videos, as well as watch students perform the skills via breakout rooms. However, any online platform with similar capabilities would suffice.

We adapted Peyton’s 4 stage teaching approach for acquisition of procedural skills, so that it was applicable for the virtual session [13]. Students were shown a skill (e.g. tying a reef knot) in its entirety via a pre-recorded video demonstration, they were split into virtual breakout rooms where the instructor deconstructed the skill; giving students the chance to ask questions and identify the next steps, the students were then supervised whilst performing the skill themselves and given real-time feedback. The process was repeated for each subsequent skill. To supervise each student in the breakout room, instructors would ‘pin’ student screens to enlarge the view of the individual undertaking the skill, give immediate feedback and ensure they could carry out the skill, before observing the next student in the breakout room. The instructors were also rotated for each breakout session whereas the student groups remained the same; to enable the group to build rapport. All students were either ‘very satisfied’ or ‘satisfied’ with the use of breakout rooms to practice the skills in a supervised environment. Instructors commented on the need for clear articulation of instructions to ensure student progression in the online teaching space, due to inability to provide tactile or other modes of feedback.

A dedicated instructor per breakout room was integral to the success of our teaching and progression of students at a similar pace. Wallace, Sturrock and Gishen [8] also used breakout rooms for procedural skills practice for medical students in their fourth year of medical school. Rather than assigning an instructor for each breakout group, tutors dropped in to troubleshoot when necessary. Emergent themes from student feedback indicated that students liked the use of breakout rooms as they were able to socialise and learn with their peers however, there were comments regarding their overuse and the slow pace of the sessions. Co and Chu [11] and Co, Chung and Chu [12] conducted a web-based basic surgical skills session with final year medical students in Hong Kong. In this session, 30 students were taught by 1 instructor and breakout rooms were not used. The authors of this paper felt this may have prevented adequate supervision and meant that training time for each individual skill took longer. If a student was having difficulty in performing a skill, the entire class would have to wait until that individual was proficient before being able to move on to the next activity.

During surgical skills breakout sessions, our instructors were communicating via a separate private messaging channel. This enabled the groups to coordinate timings and ensure the session ran smoothly. If a student was having difficulty with skills practice, the instructor was able to highlight this to the teaching team via this channel; so that when groups were rotated they could spend a little extra time with that individual. The use of breakout rooms can be time efficient especially when teaching students of differing skills and abilities however, good communication between instructors needs to be maintained throughout to optimise and coordinate their use.

Prior to the session, 40% of students ‘agreed’ or ‘strongly agreed’ that their view of the surgical skills demonstrations would be negatively impacted by holding the session online, 30% were neutral. Following the session, only 10% ‘agreed’ (0% strongly agreed) that their view was negatively impacted and 15% were neutral.

All videos and live-demonstrations on our course were performed in ‘Surgeon’s view’ (Fig. 1b). This reduces cognitive overload as the student is able to directly emulate the steps of the skill rather than needing to mentally invert the process prior to task execution. Several authors have discussed the benefits of ‘Surgeon’s view’ camera angle for teaching of surgical skills and procedures [11, 14–16]. Co and Chu [11] placed their camera device behind the instructor when demonstrating the skill live. Out of the 30 students who participated in their study, 21 rated their view of surgical knot tying demonstrations between 7-10 out of 10 (Likert scale). Bizzotoo et al. [14], Nair et al. [15] and Chao et al. [16] discuss the utility of a head-mounted, commercially available camera device for teaching surgical procedures (GoPro®). Bizzotoo et al. [14] and Nair et al. [15] used their device to record surgical procedures and then edited the videos for the purposes of teaching. Both reported head-mounted camera angles as ideal for teaching, as the field of view of the surgeon was reproduced in the video recordings. A downside however, was that when the surgeon changed positions, for instance bending or stooping, the view could be compromised [15]. Chao et al. [16] utilised live-streaming of surgical procedures to create a virtual elective during the pandemic. Students were able to interact with the operating team during the procedures, promoting learning through engagement.

Our study has some limitations. Firstly, our results are based on the online delivery of an ophthalmic surgical skills session delivered to a small student cohort. We did not directly compare online delivery with face-to-face delivery of surgical skills teaching to see if one method is superior to the other in terms of gaining skills competency. Due to the pandemic, the majority of the teaching delivered was virtual, synchronous and didactic teaching and so a virtual interactive practical skills session may have been rated more favourably by the students. To account for this, we collected pre- and post- session questionnaires. We were also not able to formally assess student competency in the skills taught to provide a more objective measure of skills attainment. The majority of students in this cohort are pursuing an ophthalmic career and therefore may be more motivated to acquire surgical skills than a more generalised audience. However, the skills taught ranged from basic (tying a reef knot) to advanced (trabeculectomy releasable suture), indicating that online delivery can be utilised to teach a range of skills to students of varying levels; including those in postgraduate ophthalmic residency training.

In conclusion, we demonstrate the successful delivery of a virtual ophthalmic surgical skills session in terms of attainment of skills and student satisfaction. Detailed preparation of teaching and high instructor to student ratios are required for success. By conducting this session online we were able to widen accessibility and participation, which has future implications for surgical skills teaching and its reach.

### Summary

#### What was known before

- Remote learning has improved equitability and accessibility of medical education.
- Online platforms have been used to deliver surgical skills teaching with varying success.

#### What this study adds

- ‘Surgeons View’ reduces cognitive overload and students found it easier to acquire skills when demonstrations were in this view.
- Breakout rooms with dedicated instructors provides individual feedback to students, improving learning outcomes.
- Clarity of verbal instructions from tutors are important when teaching surgical skills online.

## Supplementary information

Supplementary material. Post course Student Questionnaire

Supplementary material. Pre course Student Questionnaire

Supplementary material. Semi structured interview questions for Instructors
