# Supplementary material for: Development of virtual ophthalmic surgical skills training
Source: Eye (Lond). 2022 Jan 20;37(2):290–6. doi: 10.1038/s41433-021-01896-1 (PMC8773404; doi:10.1038/s41433-021-01896-1)
Supplement: Supplementary file 1 — Supplementary material. Post course Student Questionnaire [file 41433_2021_1896_MOESM1_ESM.docx]

**Microsurgical Skills Course 28.01.21 (Post-Course) Questionnaire**

**(Originally completed in Google Forms)**

Please complete this form AFTER your virtual microsurgical skills session

Name

…………………………………………………………………………………………………………………………………………………………….

I consent to the use of my answers in this questionnaire for future teaching and publication purposes. All data will be anonymised.

| Yes |  |
| --- | --- |
| no |  |

| **The following questions relate to your satisfaction of the course** |
| --- |

| THE RESOURCES. How satisfied are you with... |
| --- |

| The delivered surgical skills equipment kit? (please circle) | | | | |
| --- | --- | --- | --- | --- |
| Very Satisfied | Satisfied | Neutral | Dissatisfied | Very Dissatisfied |

| THE PLATFORM. How satisfied are you with... |
| --- |

| The Zoom Platform used to deliver the course | | | | |
| --- | --- | --- | --- | --- |
| Very Satisfied | Satisfied | Neutral | Dissatisfied | Very Dissatisfied |

| The ‘surgeon’s-view’ camera angle whilst watching surgical videos? | | | | |
| --- | --- | --- | --- | --- |
| Very Satisfied | Satisfied | Neutral | Dissatisfied | Very Dissatisfied |

| Your ability to ask questions via the Zoom platform. | | | | |
| --- | --- | --- | --- | --- |
| Very Satisfied | Satisfied | Neutral | Dissatisfied | Very Dissatisfied |

| The use of breakout rooms to practice each skill individually | | | | |
| --- | --- | --- | --- | --- |
| Very Satisfied | Satisfied | Neutral | Dissatisfied | Very Dissatisfied |

Please comment regarding your thoughts on the virtual platform, and how we can improve this further.

|  |
| --- |

| THE COURSE. How satisfied are you with... |
| --- |

| The depth of content covered. | | | | |
| --- | --- | --- | --- | --- |
| Very Satisfied | Satisfied | Neutral | Dissatisfied | Very Dissatisfied |

| The range of topics covered. | | | | |
| --- | --- | --- | --- | --- |
| Very Satisfied | Satisfied | Neutral | Dissatisfied | Very Dissatisfied |

| The balance of differing teaching methods used (lecture, video, breakout room) | | | | |
| --- | --- | --- | --- | --- |
| Very Satisfied | Satisfied | Neutral | Dissatisfied | Very Dissatisfied |

| THE FACULTY How satisfied are you with... |
| --- |

| The subject knowledge of the instructors | | | | |
| --- | --- | --- | --- | --- |
| Very Satisfied | Satisfied | Neutral | Dissatisfied | Very Dissatisfied |

| The enthusiasm of the instructors | | | | |
| --- | --- | --- | --- | --- |
| Very Satisfied | Satisfied | Neutral | Dissatisfied | Very Dissatisfied |

| The feedback given to you by the instructors | | | | |
| --- | --- | --- | --- | --- |
| Very Satisfied | Satisfied | Neutral | Dissatisfied | Very Dissatisfied |

| The ratio of students : instructors in the breakout rooms | | | | |
| --- | --- | --- | --- | --- |
| Very Satisfied | Satisfied | Neutral | Dissatisfied | Very Dissatisfied |

| THE TOPICS. How satisfied are you regarding the learning and practical skills acquired in the following... |
| --- |

| Lecture: Suture and knot-tying basics | | | | |
| --- | --- | --- | --- | --- |
| Very Satisfied | Satisfied | Neutral | Dissatisfied | Very Dissatisfied |

| Recorded video demonstrations | | | | |
| --- | --- | --- | --- | --- |
| Very Satisfied | Satisfied | Neutral | Dissatisfied | Very Dissatisfied |

| Practical 1 : Suture and knot-tying | | | | |
| --- | --- | --- | --- | --- |
| Very Satisfied | Satisfied | Neutral | Dissatisfied | Very Dissatisfied |

| Practical 2: Trabeculectomy releasable suture practice on apples | | | | |
| --- | --- | --- | --- | --- |
| Very Satisfied | Satisfied | Neutral | Dissatisfied | Very Dissatisfied |

| **The following questions relate to your confidence in the knowledge and skills acquired.** |
| --- |

| SURGICAL SKILLS |
| --- |

| I feel confident in how to correctly use and handle the instruments provided in the surgical kit | | | | |
| --- | --- | --- | --- | --- |
| Strongly Agree | Agree | Neutral | Disagree | Strongly Disagree |

| I feel confident in my basic suturing capabilities. | | | | |
| --- | --- | --- | --- | --- |
| Strongly Agree | Agree | Neutral | Disagree | Strongly Disagree |

| I feel confident in tying a reef knot and a slip knot. | | | | |
| --- | --- | --- | --- | --- |
| Strongly Agree | Agree | Neutral | Disagree | Strongly Disagree |

| **The following question relates to Covid-19 and the shift to a virtual microsurgical skills course.** |
| --- |

| Being taught online was an appropriate medium, given Covid-19 restrictions. | | | | |
| --- | --- | --- | --- | --- |
| Strongly Agree | Agree | Neutral | Disagree | Strongly Disagree |

| Do you feel the following aspects of teaching will be negatively impacted by holding this session online |
| --- |

| View of surgical simulation demonstrations (i.e. your view and orientation of the demo) | | | | |
| --- | --- | --- | --- | --- |
| Strongly Agree | Agree | Neutral | Disagree | Strongly Disagree |

| Level of supervision | | | | |
| --- | --- | --- | --- | --- |
| Strongly Agree | Agree | Neutral | Disagree | Strongly Disagree |

| Interaction with instructors | | | | |
| --- | --- | --- | --- | --- |
| Strongly Agree | Agree | Neutral | Disagree | Strongly Disagree |

| **OVERALL** |
| --- |

| How would you rate the virtual microsurgical skills session (1-10, 1: Not worthwhile, 10: Excellent) | | | | | | | | | |
| --- | --- | --- | --- | --- | --- | --- | --- | --- | --- |
| 1 | 2 | 3 | 4 | 5 | 6 | 7 | 8 | 9 | 10 |

Please comment on any aspects of the course which you thought were done well.

|  |
| --- |

Please comment on how we can further improve these virtual sessions; should the current pandemic restrictions remain and prevent face-to-face teaching.

|  |
| --- |
