# Supplementary material for: Development of virtual ophthalmic surgical skills training
Source: Eye (Lond). 2022 Jan 20;37(2):290–6. doi: 10.1038/s41433-021-01896-1 (PMC8773404; doi:10.1038/s41433-021-01896-1)
Supplement: Supplementary file 2 — Supplementary material. Pre course Student Questionnaire [file 41433_2021_1896_MOESM2_ESM.docx]

**Microsurgical Skills Course 28.01.21 (Pre-Course) Questionnaire**

**(Originally completed in Google Forms)**

Please complete this form BEFORE your virtual microsurgical skills session

Name

…………………………………………………………………………………………………………………………………………………………….

I consent to the use of my answers in this questionnaire for future teaching and publication purposes. All data will be anonymised.

| Yes |  |
| --- | --- |
| no |  |

Please state your gender

| Male |  |
| --- | --- |
| Female |  |
| Prefer not to say |  |

Have you previously attended a microsurgical skills course?

| Yes |  |
| --- | --- |
| no |  |

If yes, please indicate if this was a virtual or face-to-face course.

| Virtual |  |
| --- | --- |
| Face- to-face |  |
| Both |  |

| Regarding our communication with you: |
| --- |

| How satisfied were you with the pre-course information provided? (please circle) | | | | |
| --- | --- | --- | --- | --- |
| Very Satisfied | Satisfied | Neutral | Dissatisfied | Very Dissatisfied |

| Regarding your baseline skills: |
| --- |

| I feel confident in how to correctly use and handle the instruments provided in the surgical kit | | | | |
| --- | --- | --- | --- | --- |
| Strongly Agree | Agree | Neutral | Disagree | Strongly Disagree |

| I feel confident in my basic suturing capabilities. | | | | |
| --- | --- | --- | --- | --- |
| Strongly Agree | Agree | Neutral | Disagree | Strongly Disagree |

| I feel confident in tying a reef knot and a slip knot. | | | | |
| --- | --- | --- | --- | --- |
| Strongly Agree | Agree | Neutral | Disagree | Strongly Disagree |

| Do you feel the following aspects of teaching will be negatively impacted by holding this session online |
| --- |

| View of surgical simulation demonstrations (i.e. your view and orientation of the demo) | | | | |
| --- | --- | --- | --- | --- |
| Strongly Agree | Agree | Neutral | Disagree | Strongly Disagree |

| Level of supervision | | | | |
| --- | --- | --- | --- | --- |
| Strongly Agree | Agree | Neutral | Disagree | Strongly Disagree |

| Interaction with instructors | | | | |
| --- | --- | --- | --- | --- |
| Strongly Agree | Agree | Neutral | Disagree | Strongly Disagree |

Please comment on any specific concerns you have in being taught a microsurgical skills course online.

|  |
| --- |
