# Supplementary material for: Development of virtual ophthalmic surgical skills training
Source: Eye (Lond). 2022 Jan 20;37(2):290–6. doi: 10.1038/s41433-021-01896-1 (PMC8773404; doi:10.1038/s41433-021-01896-1)
Supplement: Supplementary file 3 — Supplementary material. Semi structured interview questions for Instructors [file 41433_2021_1896_MOESM3_ESM.docx]

**Semi-structured Interview Questions for Instructors**

**Pre session perceptions**

1. Please describe any concerns you had prior to the session

**Post session perceptions**

1. Overall, how do you feel the virtual microsurgical skills session went?
2. Did your perception change towards virtual delivery of microsurgical skills session?
3. How satisfied were you in your ability to interact and supervise students virtually?
4. Please describe (if any) adaptation to your teaching style needed to facilitate the session online.
5. How well do you feel students acquired skills in knot tying and suturing remotely?
6. Do you feel students’ skill acquisition was comparable with face to face delivery of the same session?
7. Describe what you found most challenging in the facilitation of this session?
8. What went well in the delivery of this session?
9. What are the advantages (if any) of delivering this session virtually?
10. What could be done differently to improve the session?
11. Is there a role for virtual microsurgical skills session post COVID restrictions?
